# More than a feeling: A global economic valuation of subjective wellbeing damages resulting from rising temperatures

**DOI:** 10.1371/journal.pone.0299983

**Published:** 2025-02-07

**Authors:** Stephan Dietrich, Stafford Nichols

**Affiliations:** 1 School of Business and Economics of Maastricht University and the UNU-MERIT School, Maastricht, Limburg, The Netherlands; 2 The UNU-MERIT School, Maastricht, Limburg, The Netherlands; Kwame Nkrumah University of Science and Technology, GHANA

## Abstract

When estimating economic and welfare effects from climate change, impact models must make broad assumptions because of a lack of data and the complexity of damage mechanisms. In this paper, we apply a novel approach to try and address this issue. We use an experienced utility approach to measure how extreme heat affects subjective wellbeing. The data comes from a life evaluation question collected on nationally representative surveys covering 160 countries, conducted annually for 13 years. We take advantage of 40 years of variation in daily land surface temperature data, finding that one additional exceptionally hot day significantly lowers wellbeing, by roughly 0.5% on average. This is equivalent to the wellbeing loss resulting from GDP decreasing by several percentage points. The effect size varies substantially between, and within, countries, highlighting the importance of having local data. Further, we analyze the source of wellbeing damages, by looking at income and non-income pathways. Suprisingly, we find that income pathways accounts for only a small proportion of wellbeing damages caused by extreme temperatures. This indicates current models that focus on income pathways are likely missing sources of climate-caused damages.

## 1. Introduction

Understanding the impact that climate change has on society is critical. U.S. policymakers have relied on one measure, the Social Cost of Carbon, to allocate roughly $1 trillion in government spending [1]. The Social Cost of Carbon is based on Integrated Assessment Models (IAMs), which provide laudable attempts to connect climate change effects to social and economic welfare damages [2, 3]. However, the calibration of IAMs are based on sparse data points collected from rich western countries and have very little theoretical underpinning for most of their assumptions and chosen parameters [4–6]. In this paper, we seek to quantify the effects rising temperatures have on subjective wellbeing using a different methodology, an experienced utility approach. Further our underlying survey data is globally representative, allowing us to study differences between low-income and high-income countries.

Our primary research question is: What is the effect of extreme-heat days on people’s subjective wellbeing? We also subsequently consider: How do these effects vary by population across the globe, and how do they relate to changes in levels of income?

We combine two streams of climate research. The first stream is the climate economics literature concerned with the monetization or quantification of damages caused by climate change. Economists have modeled this complex relationship for decades with increasing sophistication, in order to give policymakers tools to conduct cost-benefit analyses around the tradeoffs between economic development and adverse environmental consequences [7, 8]. To estimate climate change damage functions, a “dose response function” is modeled based on observed changes over time [9]. These functions have been made possible by greater computing power and advances in statistical causal inference theory, and have allowed economists to link rising temperatures to important social outcomes that relate directly to wellbeing, such as economic growth [10, 11], health [12, 13], conflicts [14, 15], agriculture [16], migration [17], mortality [18], crime [19], and many others (see Carleton and Hsiang for a review) [20]. The limitation of this approach, however, is that the models are not very generalizable to situations outside of the underlying data.

The second stream of research focuses on evaluating non-market and environmental goods using an experienced utility approach that is based on subjective wellbeing (SWB) [21–23]. This research uses ex-post SWB to measure intangible factors such as mental health effects [24, 25]. It has allowed researchers to estimate the value of non-monetary goods such as water quality, air pollution, and lifestyle [26–29].

We argue that current IAM models fail to fully incorporate non-market goods that affect individuals’ lives and, therefore, inaccurately value climate change damages. Missing price signals of these non-market goods make them particularly difficult to monetize. To address this shortcoming, we apply an experienced utility approach to a global database of SWB survey data from 160 countries that was collected annually for 13 years. We combine this survey data with 40 years of daily land surface temperature. A range of environmental economic papers incorporate SWB measures to value environmental public goods using an experienced utility approach [30–32]. The underlying idea of this approach is to compute the marginal rate of substitution between the marginal utility of income and the marginal utility of the environmental good, where utility is approximated by SWB reports. Consequently, the marginal benefits and costs of a non-market good can be estimated with SWB data.

Unlike previous models that assume a series of specific causal relationships between global warming and wellbeing, our approach simply looks at how wellbeing changes as people experience unusually high temperatures. The SWB construct used in this analysis is Life Evaluation, an umbrella indicator that allows respondents to consider and weigh any factor they deem important in their lives, and does not rely on researchers deciding what factors to include and calibrate [33]. This subjective measure provides a broad net with which to capture and value climate change impacts. This is an appropriate approach because the warming of the planet is a complex global natural phenomenon that affects lives in known and unknown ways.

This paper studies the impacts of rising temperatures because that is the central climate change issue addressed in the key climate policy frameworks, such as the Paris Climate Agreement. Heat is one of the most studied aspects of climate change, and researchers generally agree that it hurts wellbeing [34, 35]. The causal mechanisms are less clear though. Researchers have suggested it could be due to economic costs and the inability to work [36, 37], reduced quality or quantity of sleep [38], the associated levels of increase in crime [39], or mental health issues related to high levels of air pollution and air particulates [40, 41]. However, these studies face an important limitation: they focus on weather, not climate. *Weather* describes short-term atmospheric events, whereas *climate* describes long-term probabilistic trends observed over time. Our paper studies climate by considering the long-term underlying distributions of temperature.

We establish an empirical framework that allows us to estimate the effects of high-temperature days (HTD) on SWB, and examine how the effects differ by region and demographic group. The broad encompassing nature of our approach means it does not allow for a detailed factor analysis of specific causal mechanisms. We partially address this limitation by examining the primary determinant in SWB: income [42]. We split climate change effects on SWB into income and non-income effects, which also allows us to estimate a monetary value of these effects.

Understanding the relative size of income and non-income effects also allows us to take the analysis one step further and compute the income growth necessary to compensate for the loss of SWB caused by these increasingly frequent HTD. This provides a global model to quantify and value the damage caused to SWB by climate change, and it sheds light on the strengths and limitations of current econometric models used to value climate change’s economic damages.

Other studies have attempted to study the connection between SWB and temperature, but they face problems of geographic coverage and spatial precision. Their results suggest negative effects [43] or null effects [44–46] that in sum do not point to large effects of temperature on subjective wellbeing (SWB). However, these studies focus on industrialized countries (as do IAMs) where people’s livelihoods are less dependent on the weather. There is now a rich evidence base showing that the burden of global warming is unequally distributed, where the poor are less well-equipped to cope with harmful weather events rendering them more vulnerable to temperature changes [47–49].

This paper contributes to our understanding of how global warming affects wellbeing in several ways. First, it focuses on *climate* effects and not *weather* effects on SWB. Second, it provides global estimates that include many low-income and lower middle-income countries–some of which have never had data included in climate econometric modeling. Third, it is based on sub-national statistics which help highlight the level of variation within countries and the heterogeneity of effects. Fourth, it uses a structural equation model to differentiate between income and non-income effects on SWB, providing insight into the size of the two main theoretical causal pathways. Fifth, it uses a valuation framework to monetize these damages and helps us value them economically, which is useful from a policymaking perspective.

The remainder of the paper is organized as follows: section 2 explains the construction of the dataset and how it allows us to target sub-national regions in each country. Section 3 provides an overview of our empirical approach, where we compare SWB of respondents in the same region and year who were exposed differently to high temperatures. In Section 4, we explore the results and implications of this initial modeling, and the income and non-income effects, and examine what income growth would be required to compensate for the loss of SWB to HTD. Finally, in Section 5 we discuss our results and their implications for relevant literature and policy frameworks, and section 6 offers concluding remarks.

## 2. Data

The following section describes how we combined globally representative subjective wellbeing data with detailed daily land surface temperature data. Because the sample is drawn proportional to where people live in each country, our dataset provides a record of the temperatures human populations experienced around the world between 2008 and 2020.

### 2.1 Subjective wellbeing data

The subjective wellbeing data was obtained from the Gallup World Poll (GWP). The GWP is an annual survey conducted each year since 2006, representing 95% of the world’s population each year. Each country has a sample of N = 1,000, with the exception of a handful of high-population countries that have larger samples and a few small-population countries having samples of 500.

Our database contains information on 1.67 million interviews from 160 countries, from 2008 to 2020. Each respondent answered more than 60 questions about their life, experiences, perspectives, and basic demographics. This provided a vast interview database to include in the models.

Each country is surveyed through nationally representative, probability-based survey methods. The surveys are administered through face-to-face interviews in three-fourths of these nations and through phone interviews in the other one-quarter.

Gallup World Poll surveys are typically administered between March and October of each year, although there are several exceptions. Most interviews in our dataset were conducted in June (15%) and May (14%), and the least number of surveys were administered in January (2%) (see summary statistics in Table 1). However, in all world regions there are some large variations in survey months that cover all seasons of the year (see S1 Fig in the Appendix for an overview of survey month by region). Still, seasonality is an important consideration in the interpretation of the results. Respondents in countries that are experiencing winter while they are interviewed may report a different effect size of exceptionally high temperatures compared to a country in summer. We address this in our global model by controlling for hemisphere-specific month effects. It means that while we are not able to analyze seasonal differences in a given country, on the aggregate we can account for such seasonal differences.

**Table 1:** Summary statistics.

	N	Mean	SD	Min	Max
Subjective Wellbeing (SWB)	1671073	5.296	2.313	0	10
**Weather indicators:**					
High-temperature day (day of interview)	1670401	0.034	0.182	0	1
High-temperature days (15 days before interview)	1670401	0.403	1.332	0	15
High-temperature days (30 days before interview)	1671073	0.791	2.228	0	30
Low-temperature days (30 days before interview)	1671073	0.798	1.902	0	30
Precipitation in cm (30 days before interview)	1668243	97.559	97.757	0	1748.247
**Socio-economic characteristics:**					
Female	1671071	1.502	0.500	1	2
Primary education	1657037	0.473	0.499	0	1
Secondary education	1657037	0.424	0.494	0	1
Tertiary education	1657037	0.104	0.305	0	1
Single	1656934	0.268	0.443	0	1
Married	1656934	0.625	0.484	0	1
Separated	1656934	0.012	0.107	0	1
Divorced	1656934	0.023	0.149	0	1
Widowed	1656934	0.048	0.214	0	1
In partnership	1656934	0.025	0.156	0	1
Children	1656134	0.528	0.499	0	1
Age	1665743	39.807	16.923	15	101
Native citizen	1590070	0.977	0.150	0	1
**Month of Interview**:					
January	1671073	0.021	0.142	0	1
February	1671073	0.022	0.145	0	1
March	1671073	0.034	0.181	0	1
April	1671073	0.094	0.292	0	1
May	1671073	0.144	0.351	0	1
June	1671073	0.152	0.359	0	1
July	1671073	0.112	0.315	0	1
August	1671073	0.115	0.319	0	1
September	1671073	0.109	0.311	0	1
October	1671073	0.099	0.299	0	1
November	1671073	0.064	0.246	0	1
December	1671073	0.034	0.182	0	1
Deviation from usual month of interview	1671073	0.401	0.490	0	1
**Income:**					
Income Purchasing Power Parities (PPP)	1573899	81640	249231	0	449000
Income satisfaction	1622786	2.332	0.910	1	4

Note: Population weights were used in calculation of summary statistics.

**Fig 1:**
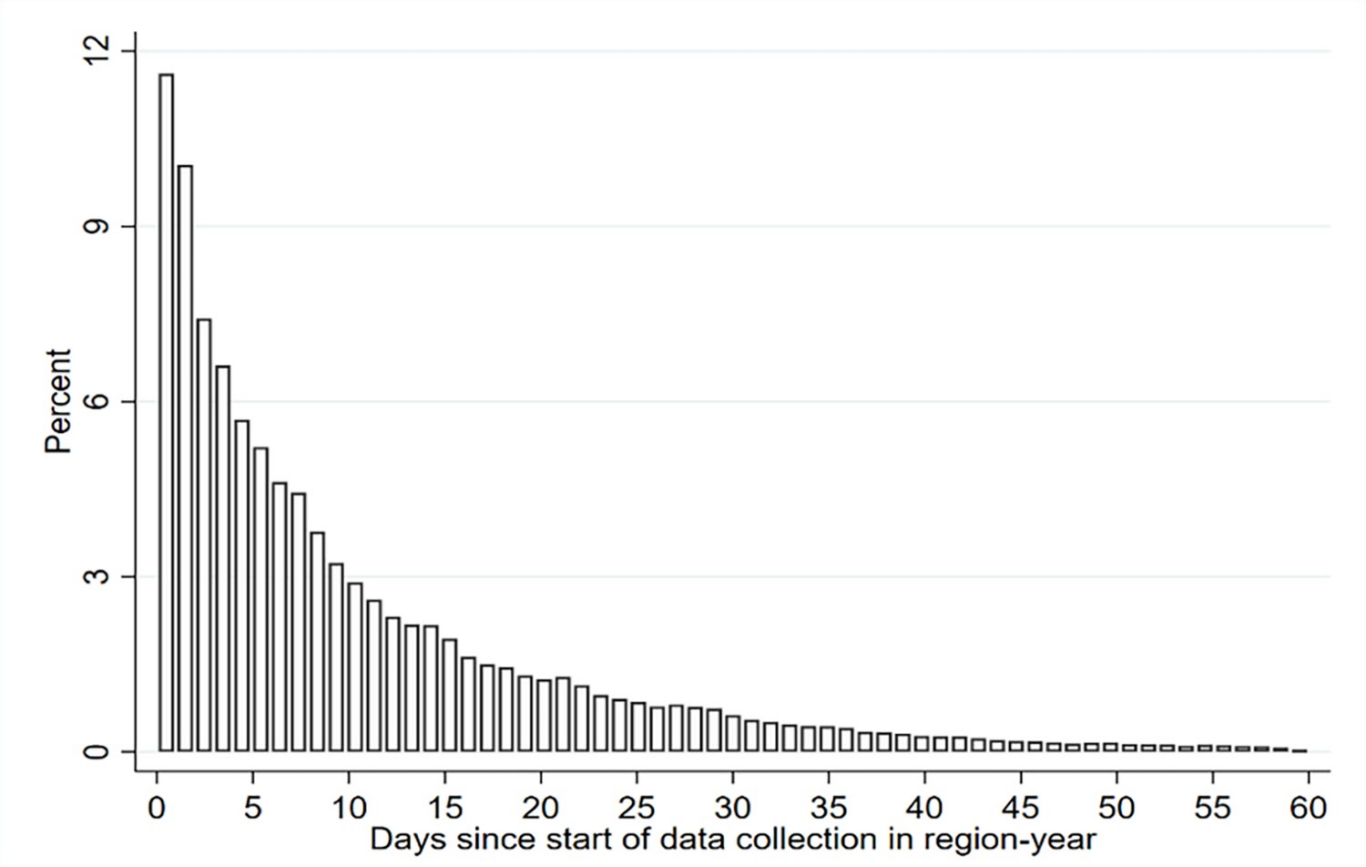
Number of days interviews were conducted after start of field work in sub-national region. Note: Days counted are in reference to the start of field work in first sub-national administrative unit.

Countries tend to be interviewed the same month every year, helping to ensure strong repeated cross-sectional data; but there are a few exceptions to this. To account for these exceptions, we control for deviations from the usual month of interview in a country. At the sub-national level, interviews are conducted in a quasi-random order throughout the region. Phone-based interviews rely on random digit-dialing sampling frames and are, therefore, randomized in terms of their timing and spatial coverage. For face-to-face interviews, the order varies with enumerator teams and selected sampling units and is based on travel logistics. Fig 1 depicts the distribution of the length of the data collection in each sub-national region and year (see S2 Fig in the Appendix for distributions by world region). As we discuss in more detail in the next section, we rely on the staggered data collection to identify the short-term effects of HTD within the empirical analysis.

Since 2008, each day that an interview was conducted has been recorded. This allowed us to match the temperature on the day of the interview, as well as each of the days preceding the interview. As discussed later, we identify the temperature 30 days before each interview. Having the date of the interview and the temperature for every day allows us to introduce variation into the dataset. For example, respondents who are interviewed at the beginning of the field period will have a different set of temperature days compared to respondents who are interviewed at the end of the field period. Most respondents, however, will share many overlapping days within the 30-day period we measure their temperature. The longer we extended the timeframe, the more days are shared between respondents and the less variation respondents have in their temperature data. This was one consideration for focusing on a short-term timeframe of 30 days.

Temperature data was merged with the survey data using the location of the first sub-national administrative level, which includes the largest sub-national boundaries, often referred to as states, provinces, departments, oblasts, etc. In total, we mapped over 3,300 administrative units to enable this spatial merge. Some administrative units cover very large surface areas, such as those in Russia’s Siberia or northern Canada. However, these units contain only a tiny fraction of the global population. In fact, the vast majority of the world’s population lives in smaller-than-average administrative units (cities and densely populated areas were often broken into smaller units to make them more governable). GWP sampling is based on each location’s relative population size within a given country. Therefore, most of the poll sample lives in administrative units with a smaller-than-average area, enabling accurate weather statistics.

#### 2.1.1 Subjective wellbeing variable

The SWB variable that serves as our dependent variable in this analysis is a Life Evaluation survey question that’s based on the Cantril Self-Anchoring Striving Scale [50]. Respondents are asked to rate their life between zero and ten based on what they consider the best and worst possible lives:

“*Please imagine a ladder with steps numbered from zero at the bottom to 10 at the top. The top of the ladder represents the best possible life for you and the bottom of the ladder represents the worst possible life for you. On which step of the ladder would you say you personally feel you stand at this time?*

There are a few characteristics of this variable that are beneficial to our analysis. First, it is a broad umbrella variable in which respondents take into account many factors of their life. Second, each respondent weights these factors according to what is most important in their life. These attributes are useful for our analysis because we are attempting to understand how climate impacts wellbeing, and the literature suggests there are a wide number of casual channels that could affect different people in different ways. Using this variable allows respondents to incorporate those factors, whether there is a known or unknown casual pathway, and establish a more accurate damage function.

The life evaluation question is the first item on the questionnaire for all 1.67 million interviews included in this analysis. There are a few notable trends to global SWB as measured by the GWP’s life evaluation question over the past 13 years. The global mean is 5.3 (see Table 1), which has slowly dropped over the years from a high of 5.45 in the beginning of the data collection to its lowest point of 5.11 in 2019.

**Fig 2:**
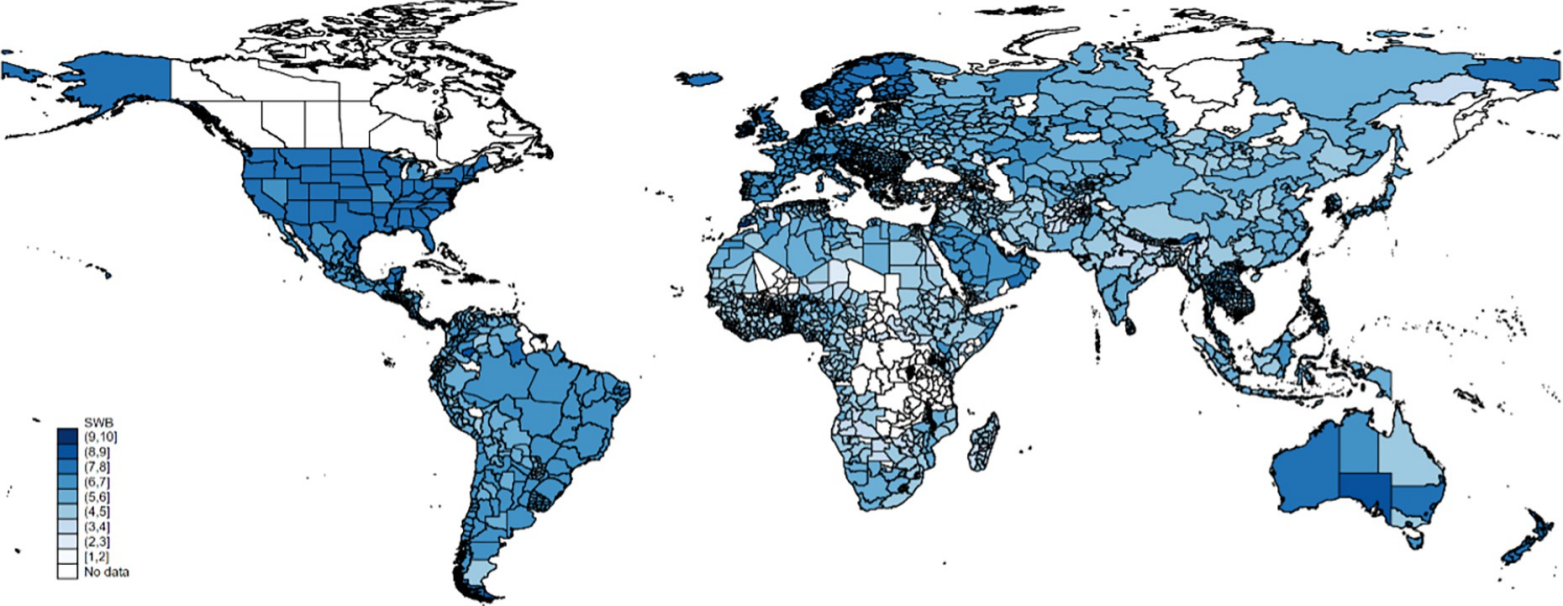
Mean life evaluation by sub-national region (2008–2020). Note: “regional” means calculated with pooled annual data of 2008 to 2020. Life evaluation is measured on a scale 0–10.

The poll’s life evaluation data has been used in many academic articles and reports, such as the United Nation’s *World Happiness Report*. In almost every case, the data is analyzed at the national level. Being able to model the data at sub-national levels improves the precision of our analysis. The mean life evaluation of each first-administrative level of each country, averaged over the last 12 years, is shown in Fig 2. It is also clear there is substantial variation within countries. For example, the life evaluation of the mountainous Indian state of Assam is 3.2, whereas the average in coastal Gujarat is 5.4. This 2.2 difference in average life evaluation represents a substantial difference in economic development levels. It would make sense that the approaches to climate mitigation policy should be different between the two states. Therefore, it is important that climate models are based on sub-national data.

#### 2.1.2 Socio-demographic variables

In our analysis, we compute the necessary income growth to compensate for the damage done to SWB by the increasing HTD. Therefore, a brief description of the income variables collected is warranted. To calculate household income, the Gallup World Poll asks respondents to report their monthly household income in local currency. If respondents have trouble answering, they are presented a set of ranges in local currency and asked to identify where they belong. A hot deck procedure imputes these cases to determine an income within the given range. Likewise, if respondents do not answer both questions, a hot deck procedure is used to impute their income. To create comparable income variables, income data is annualized and local income is converted to international dollars using the World Bank’s individual consumption purchasing power parities (PPP) conversion factor, with a 2010 base.

Besides income and SWB information, the survey collects information on a number of socio-demographic information. In Table 1, we show summary statistics of all variables used in the analysis. On average, respondents are 40 years old, half are female, 47% have a primary education, 42% have a secondary education, and 10% have a tertiary education. About 63% are married and 53% have children. The median household income is $81,640. The majority reportedly “gets by” on present income or “finds it difficult to get by”, expressed by a mean income satisfaction score of 2.3 (where a score of 1 refers to living comfortably on income and 4 refers to finding it very difficult to get by).

### 2.2 Temperature data

The temperature information was obtained from the National Aeronautics and Space Administration’s (NASA) Modern-Era Retrospective analysis for Research and Applications, Version 2 (MERRA-2) [51], a reanalysis utilizing modern hyperspectral radiance and microwave observation. This dataset contains daily temperature information (two meters above land) for the planet’s entire surface at a pixel size of about 50-by-50 kilometers. We incorporated this temperature information for every day from Jan. 1, 1980, to Dec. 31, 2020, for all land surface areas of the earth. This enabled the modeling to look at temperature changes across the planet over a 14,610-day span.

To capture the number of HTD that people face, we first established an expected distribution of temperature for every sub-national geographic region by treating the days between 1980 and 2004 as our historical baseline. It is important to note that global temperatures slowly rose throughout this period and continued to rise afterward. Consequently, our HTD means and standard deviation are based on upward sloping trends. Therefore, we treat this baseline as a rough benchmark and subsequently conducted sensitivity analysis to measure how much effect sizes change based on different HTD definitions. This timeperiod is a commonly used as a historical benchmark to measure temperature anomalies in many studies, because satellite microwave data are available starting in 1979 [52].

Next, we recorded the temperature facing respondents the day of the interview and the 30 days prior to it. In our main definition, HTD notes the total number of days before the interview that were at least two standard deviations above the historical mean, based on the sub-national geography and the time of year. For robustness tests, we varied the time period and the threshold to classify HTD.

One limitation to this approach, however, is that over a 365-day period the vast majority of temperature records are the same for respondents in the same region. Therefore, there is very little variation when comparing them over a long period of time. There is relatively more variation in a 30-day period because respondents are interviewed throughout the month. This was one reason that the hot-days variable is based on a 30-day period. This allows us to analyze short-term effects very well but means that we cannot analyze long-term effects. This is a key limitation to our approach. It is very possible the economic consequences play out over a longer period of time, and therefore we are not able to capture them using this 30-day window.

**Fig 3:**
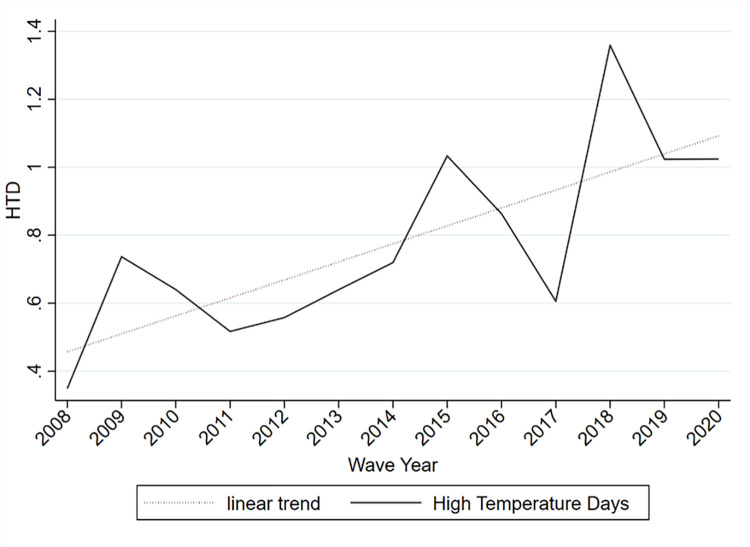
Average number of high-temperature days. Note: Population weights were used to calculate yearly means. High temperature days refer to a count of days with temperatures above 2 SD of the historical mean (1980–2004) of that period and region.

The global mean of HTD experienced by respondents in 2008 was 0.35. Over the years, the HTD became more frequent and rose to 1.02 by 2020, three times as many as in 2008. It is worth noting that 2008 was a La Nina year and cooler than average, but the data still display a large increase in HTD in a relatively short time period as illustrated by the linear trend we overlayed on the line chart in Fig 3.

Similar to Aragón, Oteiza, and Rud [53] and Deschênes and Greenstone [54], we count the number of HTD before each interview. We choose to use a relative and location-specific measure of HTD as opposed to using absolute temperature cut-offs to account for the region-specific difference of temperature extremes. That is, we want to regard how deviations from the historic mean affect SWB, and using absolute temperatures could be confounded as countries with lower temperatures tend to be richer and report higher SWB. In addition, selecting a cut-off value would become more arbitrary than the 2 SD definition that has been used in other applications to define weather extremes [55–57]. However, this definition also implies that in regions with strong seasonality and high-temperature variation, HTD tend to be less likely to occur. For an overview of mean HTD by counties’ first administrative level, refer to S3 Fig in the Appendix.

In addition, we also identified days that were below the historical mean and generated a precipitation variable using the same spatial merging technique as the temperature data. The data comes from the U.S. National Oceanic Atmospheric Administration’s (NOAA) Global Unified Gauge-Based Analysis of Daily Precipitation. As with HTD, the precipitation variable compares the 30 days before the interview to the historical mean of that same period.

## 3. Methodology

To estimate the impact of HTD on SWB, we explore the regional variation of the timing of interviews. We follow the standard empirical approach for studies on the socio-economic effects of climate with repeated cross-sectional data [58, 59]. In the main analysis, we focus on the 30 days before each interview and present sensitivity tests to alternative temperature cut-offs and recall periods as an extension. We start with a simple OLS model to estimate the average effect of high-temperature days on subjective wellbeing:

$${SWB}_{ir}=\beta_{0}+\beta_{1}HTDs_{ir}+{\beta_{2}HTD}_{ir}^{2}+\beta_{3}cold{days}_{ir}+\beta_{4}{precipitation}_{ir}+\beta_{5}C+\vartheta t+\omega r+\epsilon_{i}$$ (1)

*SWB*_*ir*_ refers to the life evaluation of respondent i in region r, and *HTD* refers to the number of high-temperature days 30 days prior to the interview. To allow for non-linear effects, we use a quadratic term of *HTD* besides the linear term. We tested the main models with cubic terms as well, but they were not statistically significant. We additionally include the number of cold days and precipitation anomalies but focus in the discussion on high-temperature days. Furthermore, we control for a vector of respondent characteristics as shown in Table 1 (except for income variables). To ensure other region and time-specific omitted variables do not confound the analysis, we gradually expand a system of time and region fixed effects that we refer to as *t* and *r* in Eq 1. The full model contains region-year and month-hemisphere fixed effects, as well as controls for country-specific time trends and deviations from the usual month of interview in a country.

In the fully expanded specification, we explore the variation in HTD among respondents in the same region-year to estimate the average effect of a unit increase in *hot days*. Thus, we compare SWB of respondents in the same region and year who were differently exposed to high temperatures. Variation in the exposure to HTD in this model originates from differences in the timing of interviews, which explains the focus on the near-term effects (the 30 days before the interview in the main analysis).

Despite the discrete and censored distribution of SWB, we use OLS models, as has been done in other research on SWB (see Maddison, Rehdanz, and Welsch for an overview [60]). This is computationally easier given the large number of observations and covariates, and it validates the results using generalized linear models that account for the censored and discrete dependent variable. In the main model, we cluster standard errors at the district-year level, the level at which our key variable of interest varies.

After estimating the effects of HTD on SWB, we predict how additional HTD affects SWB through income and non-income mechanisms. This allows us to isolate the effects of income, and estimate how much income would need to grow to compensate for additional HTD. Last, we offer a rough prediction of how much incomes would need to rise to compensate for the increasing number of HTD in the future.

## 4. Results

We focus discussion of the results on the predicted effect of a unit increase in HTD, holding all else constant at observed values. We start with a sparse model and gradually expand the number of fixed effects. Estimates of the marginal effects are presented in Table 2. For complete estimation results, refer to Table A1 in the Appendix.

**Table 2:** Marginal effect of additional hot day on SWB.

	(1) 30 Days	(2) 30 Days	(3) 30 Days	(4) 30 Days	(5) 30 Days	(6) 30 Days	(7) 30 Days	(8) 30 Days	(9) Day Interview	(10) 1–15 Days	(11) 16–30 Days
Additional hot day (2SD)	-0.013	-0.036**	-0.037**								
	(0.009)	(0.008)	(0.008)								
Hot day (binary)				-0.086**							
				(0.026)							
Additional hot day (1.5SD)					-0.017*						
					(0.005)						
Additional hot day (1SD)						-0.003					
						(0.005)					
Years 2015–2020							-0.034**				
							(0.011)				
Hot day (first 25%) (beginning of field work)								-0.047**	-0.084	-0.024	-0.102**
								(0.014)	(0.062)	(0.026)	(0.030)
Hot day (last 25%) (end of field work)								-0.060**	-0.067	-0.068**	-0.093**
								(0.016)	(0.069)	(0.022)	(0.031)
Respondent Controls	yes	yes	yes	yes	yes	yes	yes	yes	yes	yes	yes
Country-Year FE	yes	no	no	no	no	no	no	no	no	no	no
Region-Year FE	no	yes	yes	yes	yes	yes	yes	yes	yes	yes	yes
Month-Hemisphere FE	yes	no	yes	no	no	no	no	no	no	no	no
Year*Country trend	yes	no	yes	no	no	no	no	no	no	no	no

Note: Predicted marginal effects are based on OLS model with SWB as dependent variable. Standard errors are clustered at region-year level in parentheses. Controls include age, age^2^, age^3^, sex, marital status, education, children, and deviation from survey month from the usual in region. Number of observations in the first model is 1549450. The first and last 25% is used to classify the beginning and end of field work in each region-year (therefore only half the data is used in models 8–11).

* *p* < 0.05

** *p* < 0.01

The model of column 1 includes country-year fixed effects and national trends. The marginal effect estimate is negative; however, it’s not statistically significant. While the model accounts for national trends and country-fixed effects (especially in large countries), omitted local factors could bias the estimates. In column 2, we include sub-national year-specific fixed effects, the most granular geographic information available for all data waves to the model. The magnitude of the marginal effect increases compared to the previous estimate and turns highly statistically significant. The coefficient suggests that an additional HTD reduces SWB by about 0.036 points, holding all else constant at observed values. This may sound little, but it implies that a single additional high-temperature day reduces SWB by more than 0.5% in the global model. Adding additional fixed effects to account for month and region-specific effects, as well as country trends, does not change this finding (column 3). The effect is also robust to clustering standard errors at the country level instead of the region-year level and to using a censored generalized linear model with a poisson link function that accounts for the discrete distribution (see S4 Fig in the Appendix for the effects by country).

Often, high-temperature days occur as part of heat waves that extend over several days. In column 4, we use a binary indicator that measures the occurrence of high-temperature days instead of the count of days. The results show an even stronger effect of -0.086 if at least one HTD occurred 30 days prior to the interview. It suggests the effect on the extensive margin is more impactful than on the intensive margin.

In addition, we test how applying less extreme definitions of high-temperature days affects results. In columns 5 and 6, we use 1.5 SD and 1 SD from the mean instead of the 2 SD as cut-offs to classify HTD. The coefficients are halved in magnitude if a cut-off value of 1.5 SD is used, and they turn statistically insignificant for the 1 SD definition. This implies the effects are driven by the occurrence of particularly extreme temperature days.

Furthermore, we test whether the effect changes over time, for example, because of successful adaptation to increasing temperatures. In column 8, we show the effect for the subset of data collected in the years 2015 through 2020, which represents approximately half of the dataset. The predicted marginal effect of -0.034 is not statistically different from the overall marginal effect estimate of -0.036, suggesting that effects have not decreased compared to earlier years in the database. This result also holds if we use shorter time periods or year-specific predictions, which does not point at noteworthy adaptation processes in the global data.

Next, we examine differences in effects between respondents that were interviewed in the beginning of the regional field work (first 25%) or the end (last 25%). Respondents that were interviewed later were exposed to the same HTD as early respondents; they just happened further in the past. In contrast, late respondents can be exposed to HTD that occurred after early respondents were already interviewed. Therefore, if respondents do not or only slowly recover from HTD, we would expect the HTD effect to be larger for late respondents. However, the results of column 8 do not show significant differences in effects, which could point to fast recovery processes in SWB after temperature extremes occurred. This finding also holds if we only focus on regions where the field work lasted for at least 30 days.

In the main results we use a recall period of 30 days to quantify the short-term effects of high-temperature days. A mid- or long-term evaluation perspective is complicated because identification relies on variation in the timing of interviews. To better understand the temporal dynamics, we re-estimate the model using different recall periods. The effect of a HTD on the day of the interview has a negative sign, but it is not statistically significant (column 8). It turns significant for the period from 1 to 15 days and further increases for the period from 16 to 30 days before the interview.

These results seem to suggest that the negative effects do not unfold immediately. What is driving the negative effects then? Conceptually, different mechanisms could be at play, including income or wealth effects, because of high temperatures (reduced productivity), health effects (cardiovascular, respiratory disease, or cerebrovascular diseases), or belief updates regarding future states of the world (less optimism because of experienced signs of global warming [61]. To get a sense of possible drivers of the impact, we estimate the effects of HTD on proxy indictors for wealth (income), health (subjective health problems), and SWB in five years (see S3 Table in the Appendix). The results show a negative income effect of HTD on the day of the interview that subsides after 15 and 30 days after the interview, no health effects, and negative effects on expected future wellbeing. This result could suggest that income effects occur in the short term, which may contribute to less optimistic views about wellbeing in the future and that the global impacts are not significantly driven by health effects.

To account for contextual differences, we consider effect differences by geographical region. In S4 Table in the Appendix, we present estimates by world regions. The results indicate that the global estimate is driven by negative effects in South and East Asia, where we find effect sizes of -0.132 and -0.062, respectively. Effects in Latin America, the Middle East and North Africa (MENA) region, Sub-Saharan Africa, and the Commonwealth of Independent States (former USSR states) reach about -0.03 but fail the 5% significance level and we find no effects in Europe, North America, and Australia. What explains these regional differences? One explanation is that we use a relative measure that depends on the variation of temperatures in the region. In regions with large temperature variation because of seasonality or weather phenomena such as the monsoon, HTD require a larger increase in absolute temperature than in regions with less temperature variation. That means in South Asia the impact of an additional HTD is large, but at the same time HTD are less likely to occur compared with other regions (see spatial distribution of HTD in S5 Fig in the Appendix).

In addition to world regions, we also separately estimate effects by age, gender, and wealth of respondents (see S4 Table in the Appendix). We find that respondents older than 65 are more strongly affected than younger respondents, and richer respondents (rich within countries) are more strongly affected in absolute terms but not in relative terms, and we find no gender differences on average. Breaking these estimates further down by world region shows that mechanisms differ and the effects depend on the context. Yet the results indicate that effects are largest in lower- and upper-middle income countries. Respondents in richer countries seem, on average, to be less affected by the occurrence of high-temperature days. As high-income countries are more likely to be found in cooler regions, we also tested for differential effects by countries were HTD were more likely to occur (more than 15 days above 35 degrees per year). Effects of HTD are larger in hotter (0.053) than in colder (0.031) countries, but the difference is not statistically significant. More generally, we observe that effects are driven by temperature extremes in hotter months. The effect of HTD is about twice as large if it happens in the hottest months of the world region as compared to other months with marginal effect estimates of -0.053 compared with -0.031, respectively (see S5 Table in the Appendix).

In summary, we find a statistically significant effect of HTD on subjective wellbeing that requires some time to fully unfold, but at the same time it does not last much beyond the usual field data collection period of around one month. The effect is driven by respondents in poorer countries and temperature extremes in generally hotter months. Whether and how economically meaningful these effects are is the subject of the next section.

### 4.1 Income versus non-income effect mechanisms

The results give an idea of how the increasing occurrence of temperature extremes affects wellbeing in the short run. Building effective policy interventions to manage the climate crisis requires monetizing these damages. However, missing price signals of non-market goods make it particularly difficult to monetize them. A range of environmental economic papers use SWB measures to value environmental public goods using an experienced utility approach [62–65]. The underlying idea of this approach is to compute the marginal rate of substitution between the marginal utility of income and the marginal utility of the environmental good, where utility is approximated by SWB reports. Consequently, the marginal benefits and costs of a non-market good can be estimated with SWB data. In this section, we apply the approach to compute the income increase necessary to compensate for the SWB damage caused by HTD. In a second step, we extrapolate the expected HTD in the coming decade based on the data, which we then use to calculate the income growth necessary to compensate for damage related to the projected increases in HTD.

**Fig 4:**
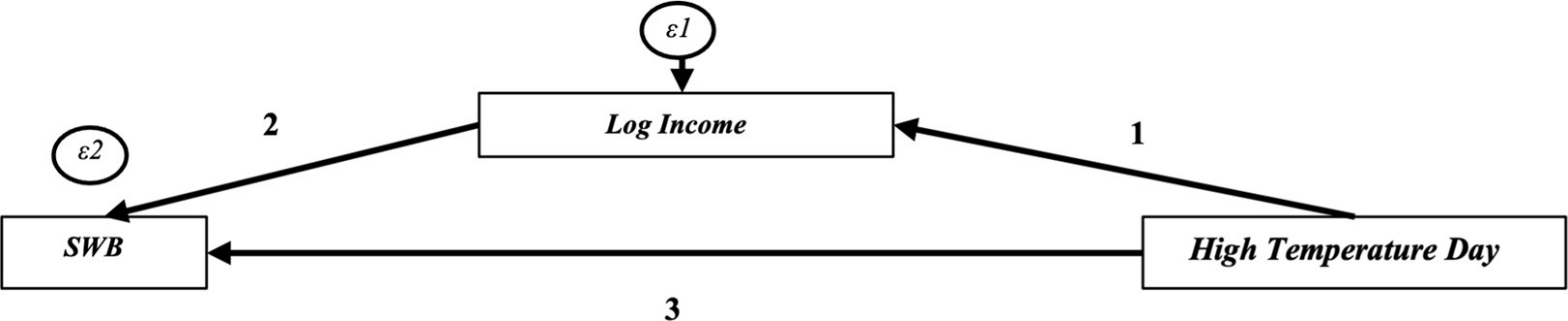
Structural model outline.

To quantify the costs, we need to understand the income and non-income damage caused by HTD. To this end, we fit a linear structural equation model as displayed in Fig 4. HTD is the main exogenous variable in the model that affects respondents’ income, for example, through production losses (1). In turn, changes in income affect SWB, as has been widely established in the literature [66–68]. We refer to this path as the income effect (1*2). As the effect of income on SWB is not linear and has been found to rather follow a logarithmic function [69,70], we log-transform income reports. The non-income mechanism is the collection of all other effects that are not captured by the income effect (3). This may include health effects, stress, psychological effects, or social conflicts, just to mention a few. The linear model includes the same control variables as the model in column 3 of Table 2, except the second equation of the income effect path also includes respondents’ income.

The predicted marginal effects are presented in Table 3, and the complete estimation results are shown in S2 Table in the Appendix. In the global model, most of the effect of HTD on SWB is explained by non-income effects, and only less than 5% of the non-income effect is related to income changes. This is because HTD only have a small effect on incomes, at least in the 30-day period considered here, which despite the very strong effect of incomes on SWB only leads to a small income effect. The point estimate of the non-income effect is almost identical to the previous estimates, which suggests the short-run effect is dominated by non-income aspects.

**Table 3:** Marginal effect of HTD on income and SWB.

	(1)	(2)	(3)	
	HTD -> Income	Income -> SWB	Non-Income	HTD Cost of Income
**Global**	-0.003	0.410	-0.035	8.8%
**E.U.**	-0.004	0.512	-0.013	2.9%
**Rest Europe**	-0.016	0.553	0.010	0.2%
**Commonwealth**	-0.004	0.437	-0.030	7.3%
**Australia**	0.005	0.344	0.009	-3.1%
**Southeast Asia**	-0.002	0.347	0.013	-3.5%
**South Asia**	0.013	0.483	-0.136	26.9%
**East Asia**	-0.016	0.487	-0.058	13.5%
**Latin America**	0.002	0.334	-0.030	8.8%
**North America**	0.012	0.262	-0.011	3%
**Mena**	-0.011	0.420	-0.015	4.7%
**Sub-Saharan Africa**	-0.010	0.227	-0.021	10.3%

Linear structural equation model, including fixed effects as in model 3 of Table 2. Estimates show marginal effects at observed values. HTD cost of income shows approximated income growth to compensate for HTD damage (1+(3/2)*100).

Two shortcomings of this approach is the short-term focus, and the negative income effects of HTD may not unfold immediately. For example, if heat damages a farmer’s crops, the income effect may not manifest until later in the year. As a robustness test, we use respondents’ income satisfaction, which we regard as a broader measure that may also encompass expected future incomes (see results in S6 Table in the Appendix). The income effect increases markedly if we use income satisfaction instead of income and reaches about 29% of the non-income effect in the global model. We regard this as an indication that expected incomes and a more long-term perspective would increase the weight of the income effect mechanism markedly; however, non-income effects remain the dominant mechanism.

Based on the structural model estimates, we can compute the income increase necessary to compensate for the SWB damage of an additional HTD. The compensation needs to cover the income damage of HTD plus the income increase to compensate for the non-income effect. As we log transformed incomes, we interpret the compensation as an approximation of percentage changes in incomes. In column 4 of Table 3, we present the average income increase needed to compensate for an additional HTD. According to the global model, it requires an 8.8% increase in income to compensate for the damage of an additional HTD, holding all else constant at observed values. This constitutes a substantial increase and helps put the main results into perspective.

As expected, the results differ largely by world region. In the lower part of Table 3 we show estimates separately by world region. The non-income effect is largest in South Asia (-0.136) and the income effect is largest in the MENA region. The effect of income on SWB is lowest in Sub-Saharan Africa and largest in non-European Union (EU) Europe. Combining those numbers, South Asia shows by far the largest “cost” of a HTD, with 26.9% of income followed by East Asia, Sub-Saharan Africa and Latin America. On the other end, in Australia and Southeast Asia signs are negative. These numbers illustrate the large discrepancy in damages that has also been found in other studies. For example, climate change is expected to increase the overall global mortality rate, but it is expected to decrease the mortality rate for northern latitudes, starting at roughly the 45^th^ parallel [71].

### 4.2 Future damages resulting from rising temperatures

**Fig 5:**
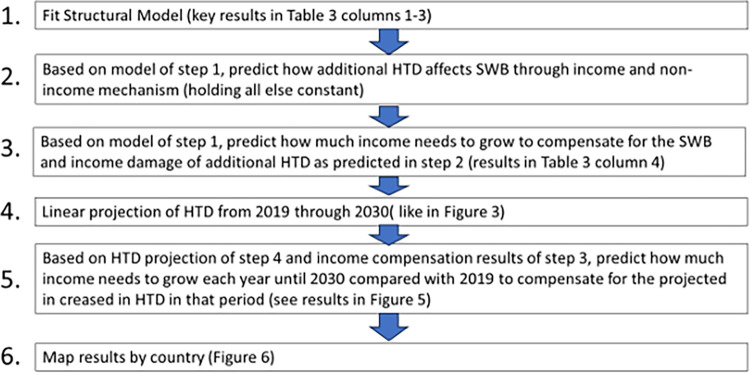
Estimation procedure flow-chart.

In the last step, we use the estimates to project income growth that will be necessary to compensate for the rising frequency in HTD. Therefore, we use a simple linear trend as shown in Fig 3 to extrapolate the increase in HTD. Slightly more sophisticated models that better capture non-linear functional forms led to similar results, but it is important to note that our goal with this exercise is not to provide the most accurate forecasts of HTD—it’s to illustrate the damage magnitude such changes may cause and how the damage is distributed. We use these estimated trends to predict HTD for each year and region for the years 2021 until 2030, and given the model simplicity, we refrain from doing more long-term projections. Thereafter, we feed the predicted HTD into the structural model to simulate the SWB damage caused by the projected changes in HTD. We calibrate the model with data from 2019, the latest survey before the pandemic. In other words, we predict how SWB changes if HTD increase (holding all else constant at 2019 values) and compute by how much incomes will need to grow to offset SWB damage considering the income and non-income effect mechanisms. A flow-chart of these steps is provided in Fig 5 to illustrate the approach.

**Fig 6:**
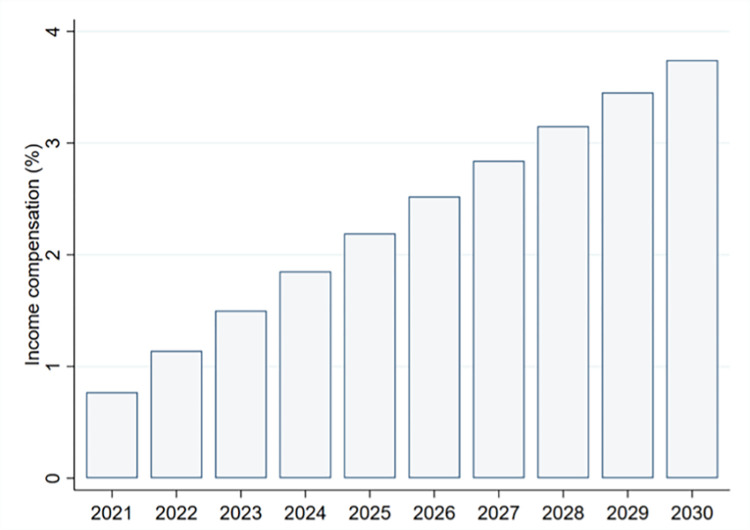
Income growth to compensate for HTD damage compared with 2019. Note: Income growth necessary to compensate for income and non-income damage of projected increase in HTD compared with 2019. Damage estimates are based on the results of the global structural equation model. The increase in HTD is extrapolated with the region-specific linear trend from 2008 to 2019. Mean compensation rates are calculated using population weights.

Fig 6 shows results of the global model indicating that, compared with 2019, it will require a growth of almost 4% in incomes by 2030 to compensate for the increase in HTD. Growth rates increase by about 0.4 percentage points yearly, and the rates (only marginal) subdue over time due to small decreasing marginal damage of an additional HTD. Using slightly more flexible functional forms to model the growing increase in HTD increases the effect. For example, using a quadratic function form instead of a linear trend increases the compensating growth to more than 5%.

**Fig 7:**
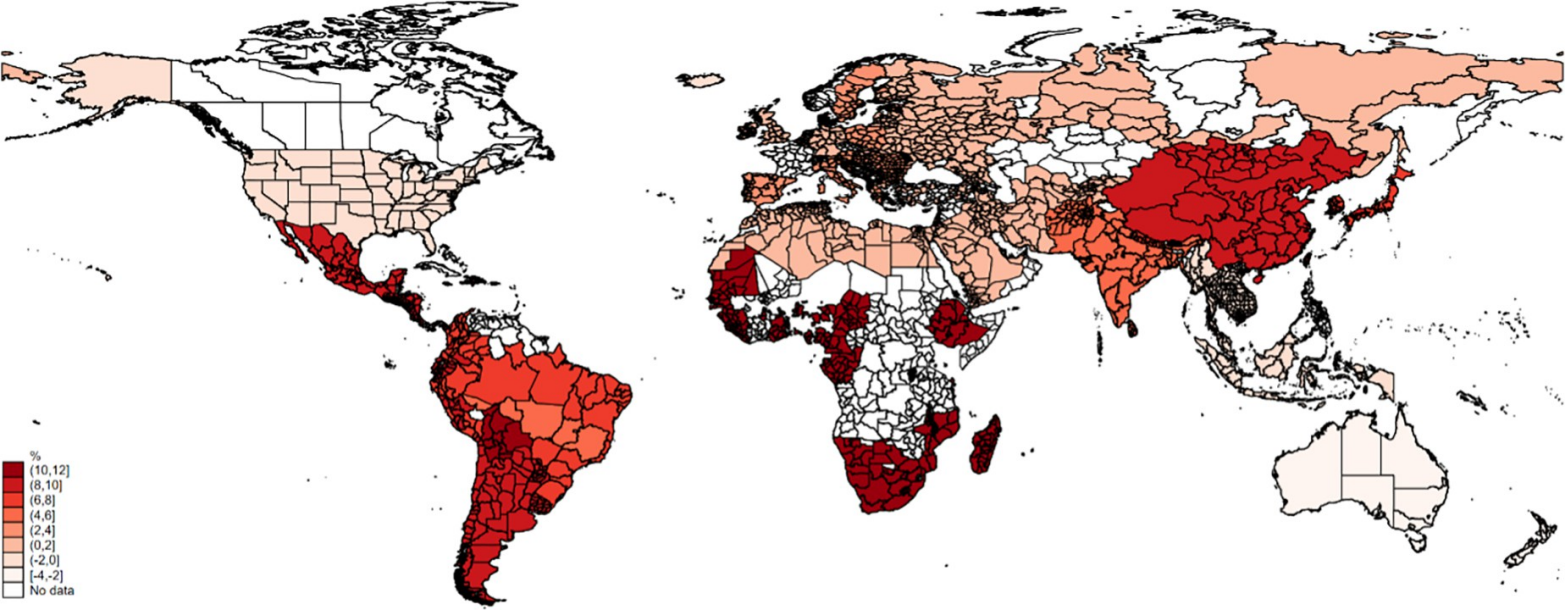
Income increase to compensate for SWB damage of increasing HTD in 2030 compared with 2019. Note: Predictions are based on region-specific linear projections of HTD changes from 2019 to 2030 and world-region specific estimates of the structural equation models.

The average compensation rates are high, but previous results suggest they are unequally distributed. In the last step we compute regional compensation rates by allowing HTD trends and SWB effects to differ regionally. We map the compensation in Fig 7. The darker the shade of red, the larger the income growth necessary to compensate for the projected SWB damage. The map clearly shows that poorer countries require disproportional growth rates than richer countries. While most high-income countries have estimated compensation rates close to or even slightly below zero, rates in Sub-Saharan Africa, South and Southeast Asia exceed 10%. With the previous results in mind, it may look surprising to see the largest rate in Sub-Saharan African countries compared with India, where we saw the largest marginal damage, but this is again mainly explained by two components. First, HTD are rather uncommon and more unlikely to occur in India than in many other regions possibly related to the large standard deviation in temperatures. This makes the marginal damage quite large but also quite unlikely. Secondly, in Sub-Saharan Africa it requires the largest increase in income to compensate for a unit reduction in SWB. This means that within our framework, the “price” for changes in SWB is about twice as high as in South Asia. To illustrate these underlying mechanisms, we show the projected change in HTD and the predicted SWB damage by world region in S6 and S7 Figs in the Appendix.

## 5. Discussion

Our results help confirm assumptions inferred by previous, more geographically limited studies. Indeed, extreme temperatures measurably lower people’s wellbeing in most countries across the world. However, the effect size varies substantially between and within countries. Our results show that the majority of the damages from rising temperatures affect people through non-market channels. These channels should be further investigated and deserve more attention in current climate econometric modeling. Our experienced utility approach used to monetize this damage estimates that growth of 4% in incomes will be necessary by 2030 to compensate for the increase in HTD.

This effect size is in line with other studies that estimate the economic damages of extreme heat [72–75]. The fact that these other studies use different methodologies to arrive at roughly the same damage sizes offer support of the experienced utility approach as a useful methodology climate researchers should explore.

Perhaps more important than the exact size of the estimated losses is our finding that income only makes up a small share of the total HTD damage on SWB. It is possible that this underestimates the income effect size because of the short-term nature of the temperature variable (only 30 days long). Nonetheless, it presents evidence that climate change is affecting people through many non-income pathways that are unaccounted for. This suggests the income-oriented economic valuations of climate-related welfare damage functions are potentially missing key variables, and that highly utilized and impactful policy instruments such as the Social Cost of Carbon and carbon emissions markets substantially undervalue climate-related damages.

### 5.1 Limitations

Although our analysis shows the rough magnitude of effects over the rest of the decade, it is important to note that this does not imply that continuous GDP growth will compensate for all HTD damages in the future. There are multiple reasons for this. First, this analysis treats HTD as exogenous. However, literature has long established a connection where GDP growth leads to more CO2 emissions [76]. If countries simply push for ever greater GDP growth to compensate for SWB damages, that GDP growth will lead to an increase in CO2 emissions, which in turn will lead to greater climate and HTD damages. This raises a second consideration: income is not a perfect substitute for SWB. While most studies show that income is the largest factor in SWB, it is only one of many factors and has diminishing marginal benefits to SWB [77]. For example, increasing HTD could lead to adverse health outcomes and mortality rates, which no increase in income would compensate for. Lastly, it should be noted that we present point estimates of results that are uncertain, and in some regions the damage estimates fail statistical significance. More precise, geo-localized SWB data collected over time would help improve the accuracy of these estimates.

Moreover, our projection model does not take into account the ability of populations to adapt to rising temperatures. Adaptation is an important consideration in climate econometrics models, and not including it can lead to an overestimation of damages to social outcomes. Evidence suggests that populations experiencing repeated extreme climate events learn to mitigate against its damages. For example, countries hit by typhoons more often report less adverse marginal GDP effects than those hit less frequently [78]. Estimated mortality impacts from climate change that do not take into account adaptation tend to overestimate impacts by a factor of 2.6 [79]. However, adaptation is difficult to quantify as it is largely unobservable. Also, the local population needs to pay for these adaptations, so it is important to take its costs into account. These costs can be substantial; they are projected to rise over time and may be unaffordable to lower-income populations. In our global model, we find that the effect size of HTD is largely the same from 2015 to 2020 as it is for the rest of the period of observation. This suggests people have not made a substantial improvement in their ability to adapt to HTD on a global level in the past, because we do not see a reduction in the HTD effect. Still, further research in this area should incorporate adaptation models to more accurately predict the future effects of sizes on SWB.

## 6. Conclusion

Overall, this paper makes several contributions to the literature. It analyzes one of the most globally comprehensive models, providing useful insight into the differences between high-income and low-income regions of the world. It demonstrates the substantial variation of effects of rising temperatures on SWB at sub-national levels as well, illustrating the importance of taking local conditions into account in any climate econometric model. Using novel methods, this paper quantifies the welfare damage of global warming’s effects around the world and is able to disentangle the income effects and non-income effects, showing, at least in the short run, the non-income effects are much higher than what is assumed in current models.

Oftentimes, the debate of climate policy is framed as a tradeoff between economic gains and environmental regulation. However, by monetizing SWB damages, we see that climate damages to society are mounting, making the economic growth required to compensate for SWB losses increasingly high and harder to obtain. The cost-and-benefit analysis that policymakers need to make around the world when evaluating environmental impacts is becoming increasingly tipped toward the side of environmental protection. This research, and future research utilizing these methods, can help policymakers understand the nuanced effect climate change has on a wide number of non-market goods across different geographies and populations. It suggests that subjective measures could be a powerful addition to climate impact models.

## Supporting information

S1 Fig Month of interview by world region. (PNG)

S2 Fig Length of data collection in district-year by world region. (PNG)

S3 Fig Mean HTD by first administrative level. Note: region means calculated with pooled data 2008–2020.
(PNG)

S4 Fig Predicted marginal effect of HTD by country. Note: Predicted marginal effects based on OLS model 3 of Table 2 with additional interaction term of country and HTD.
(PNG)

S5 Fig HTD 30 before interview by world region and survey year. Note: world region—year means calculated using population weights. HTD refer to the period 30 days prior to interview.
(PNG)

S6 Fig Projected change in SWB by world region due increasing HTD (benchmark 2019). Note: Predictions based on projected changes in HTD and world region specific structural equation models as in Table 3.
(PNG)

S7 Fig Projected change in HTD (2021–2030). Note: Projections based on linear model with region-specific trends. Figures show local polynomial smoothing with rule of thumb bandwidth of 1.
(PNG)

S1 Table Complete estimation results *(Table 2, HTD marginal effects)*. Standard errors in parentheses clustered at region-year level. * *p* < 0.05, ** *p* < 0.01, *** *p* < 0.001.
(TIF)

S2 Table Complete estimation results *(Table 3, structural model)*. Standard errors in parentheses * *p* < 0.05, ** *p* < 0.01, *** *p* < 0.001.
(TIF)

S3 Table Effect of HTD on income, subjective health, and SWB in five years. (TIF)

S4 Table Marginal effect of additional hot day by region and respondent characteristics. Predicted marginal effects based on OLS model. Standard errors clustered at region-year level in parentheses. Bold numbers refer to statistically significant differences at 5% between pairwise predictions (male-female, older-younger, or poorer-richer). * *p* < 0.05. ** *p* < 0.01.
(TIF)

S5 Table Effect by world bank income classification. (TIF)

S6 Table Income satisfaction measure. (TIF)
