# Supplementary figures and images for: More than a feeling: A global economic valuation of subjective wellbeing damages resulting from rising temperatures
Source: PLoS One. 2025 Feb 7;20(2):e0299983. doi: 10.1371/journal.pone.0299983 (PMC11805375; doi:10.1371/journal.pone.0299983)

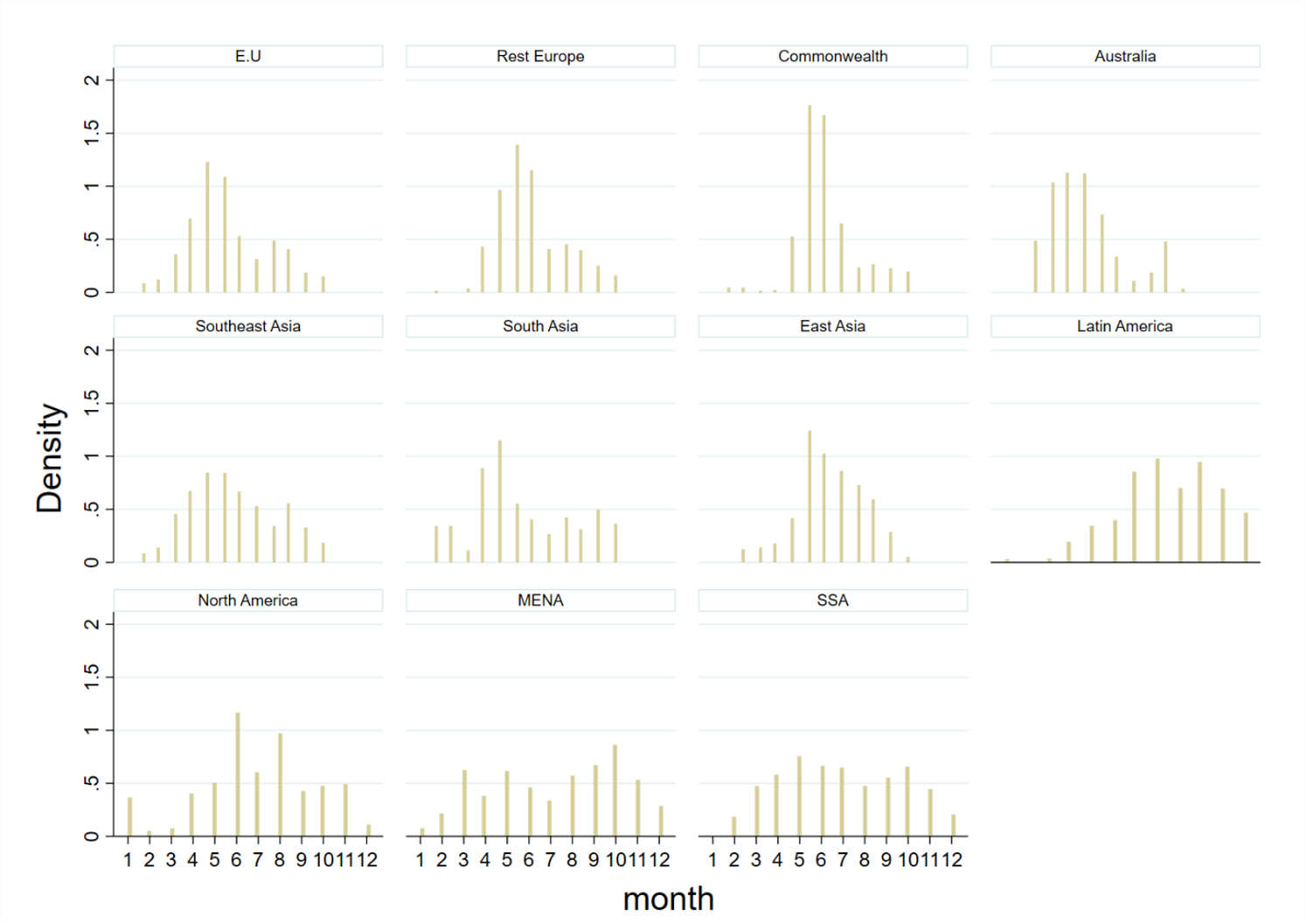

Supplement: S1 Fig — (PNG) [file pone.0299983.s001.png]

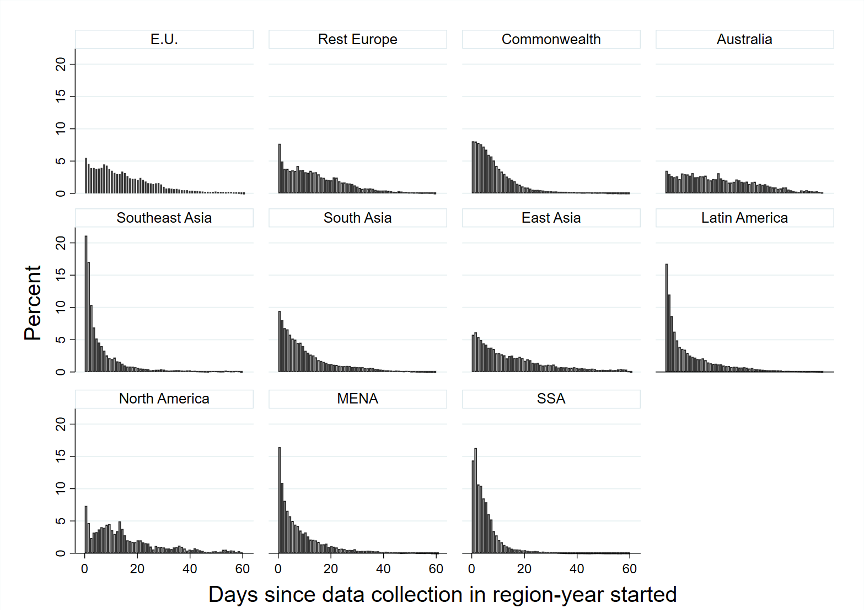

Supplement: S2 Fig — (PNG) [file pone.0299983.s002.png]

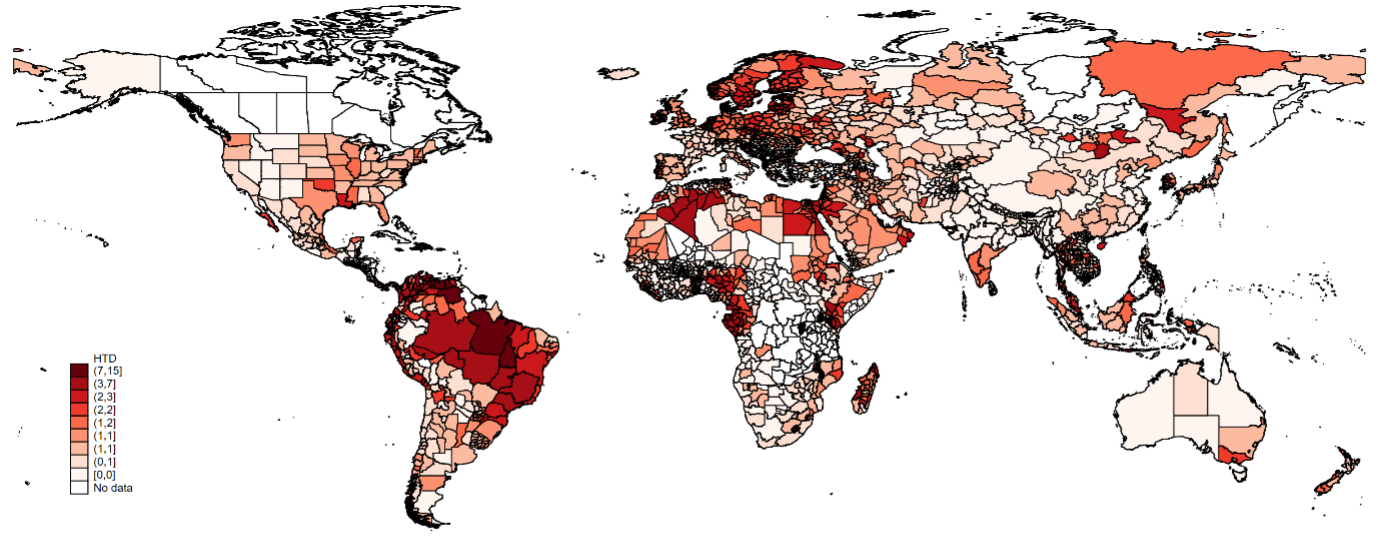

Supplement: S3 Fig — Note: region means calculated with pooled data 2008–2020. (PNG) [file pone.0299983.s003.png]

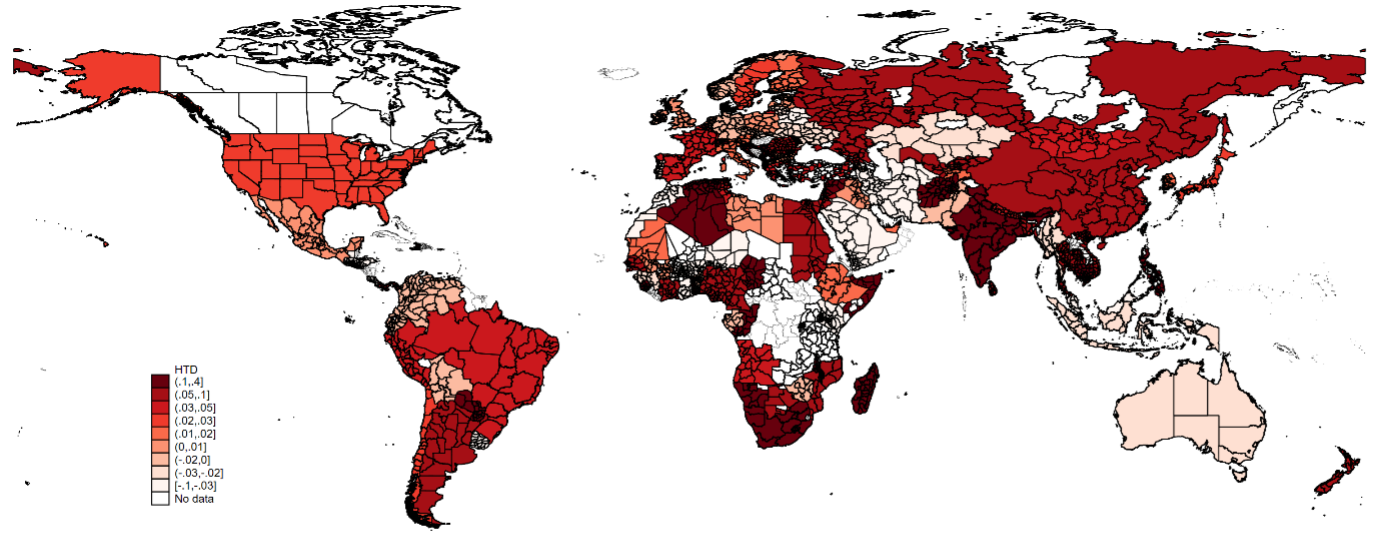

Supplement: S4 Fig — Note: Predicted marginal effects based on OLS model 3 of Table 2 with additional interaction term of country and HTD. (PNG) [file pone.0299983.s004.png]

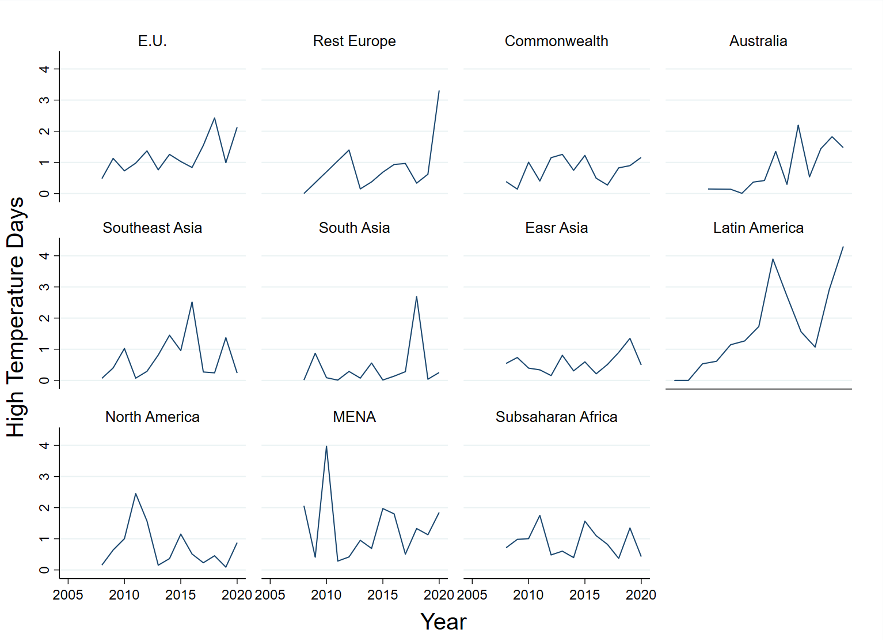

Supplement: S5 Fig — Note: world region—year means calculated using population weights. HTD refer to the period 30 days prior to interview. (PNG) [file pone.0299983.s005.png]

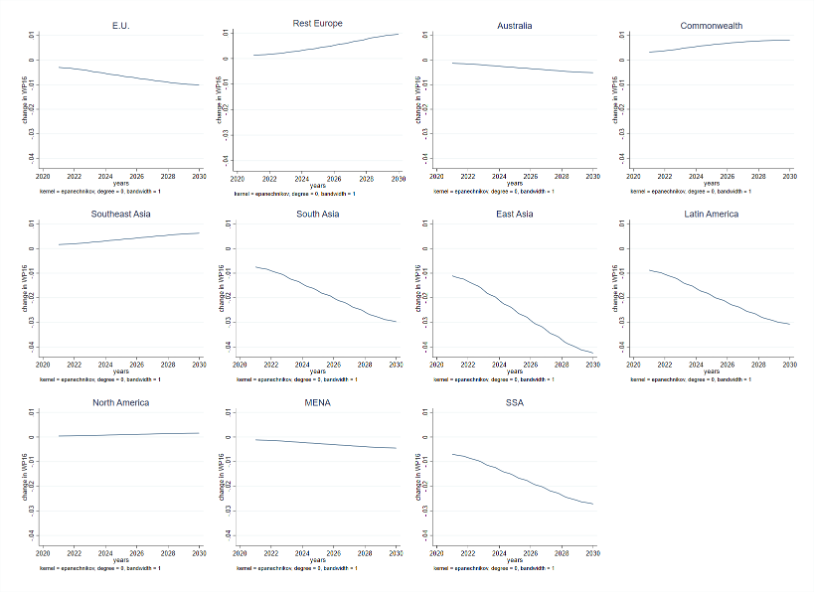

Supplement: S6 Fig — Note: Predictions based on projected changes in HTD and world region specific structural equation models as in Table 3. (PNG) [file pone.0299983.s006.png]

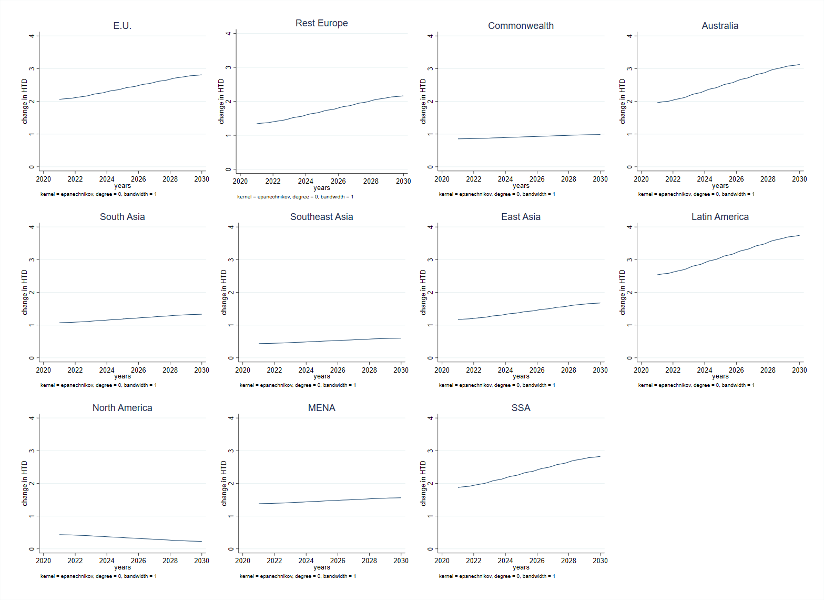

Supplement: S7 Fig — Note: Projections based on linear model with region-specific trends. Figures show local polynomial smoothing with rule of thumb bandwidth of 1. (PNG) [file pone.0299983.s007.png]

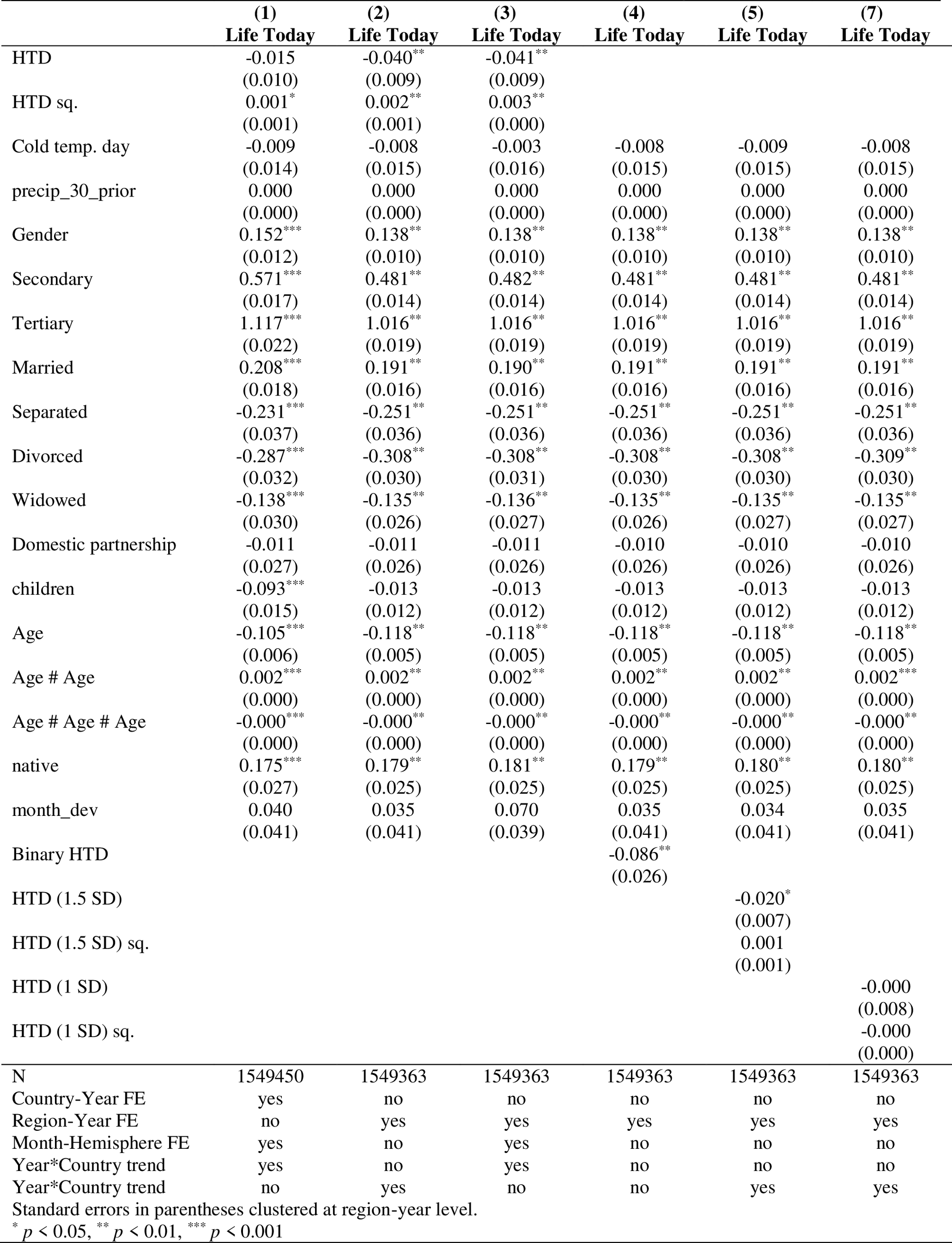

Supplement: S1 Table — Standard errors in parentheses clustered at region-year level. * p < 0.05, ** p < 0.01, *** p < 0.001. (TIF) [file pone.0299983.s008.tif]

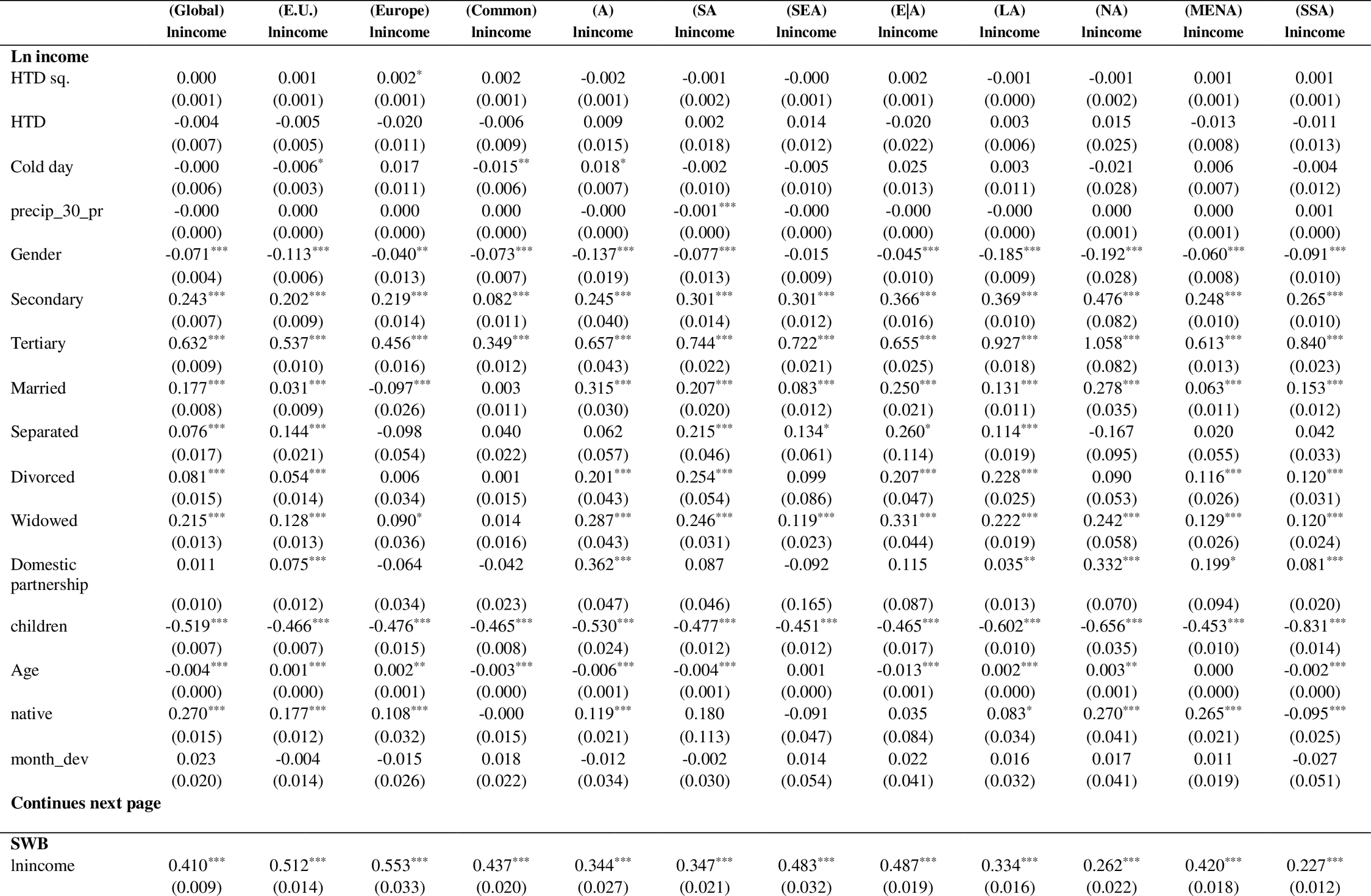

Supplement: S2 Table — Standard errors in parentheses * p < 0.05, ** p < 0.01, *** p < 0.001. (TIF) [file pone.0299983.s009.tif]

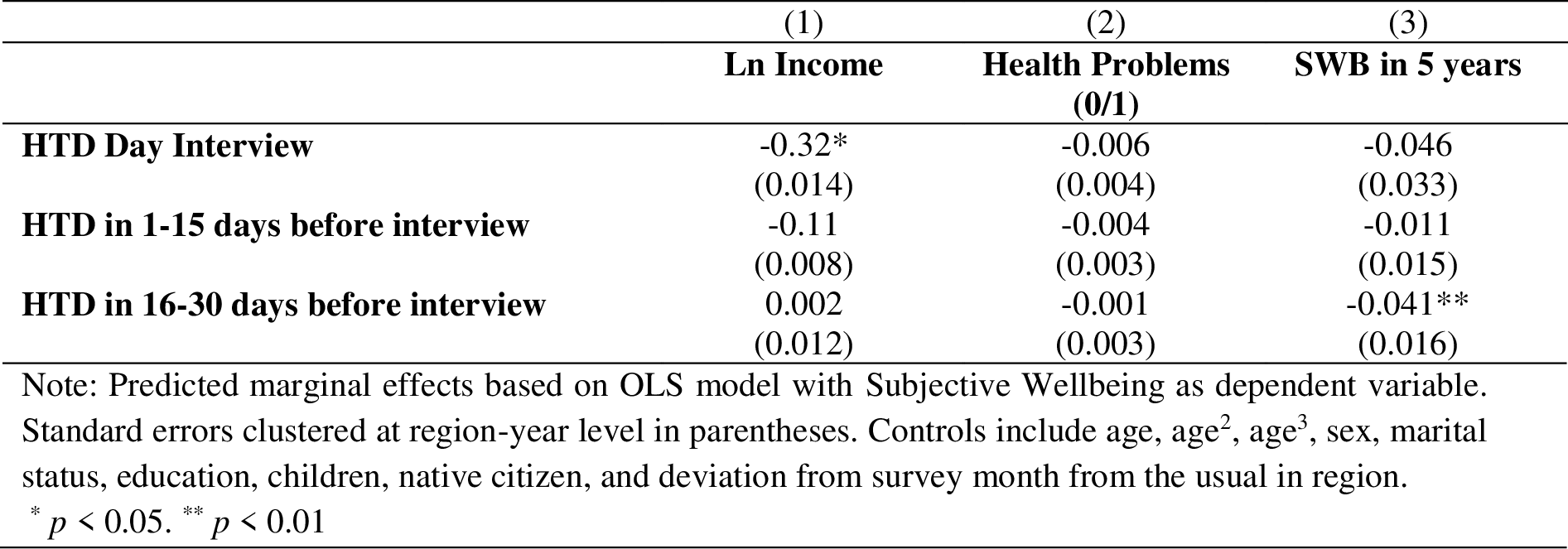

Supplement: S3 Table — (TIF) [file pone.0299983.s010.tif]

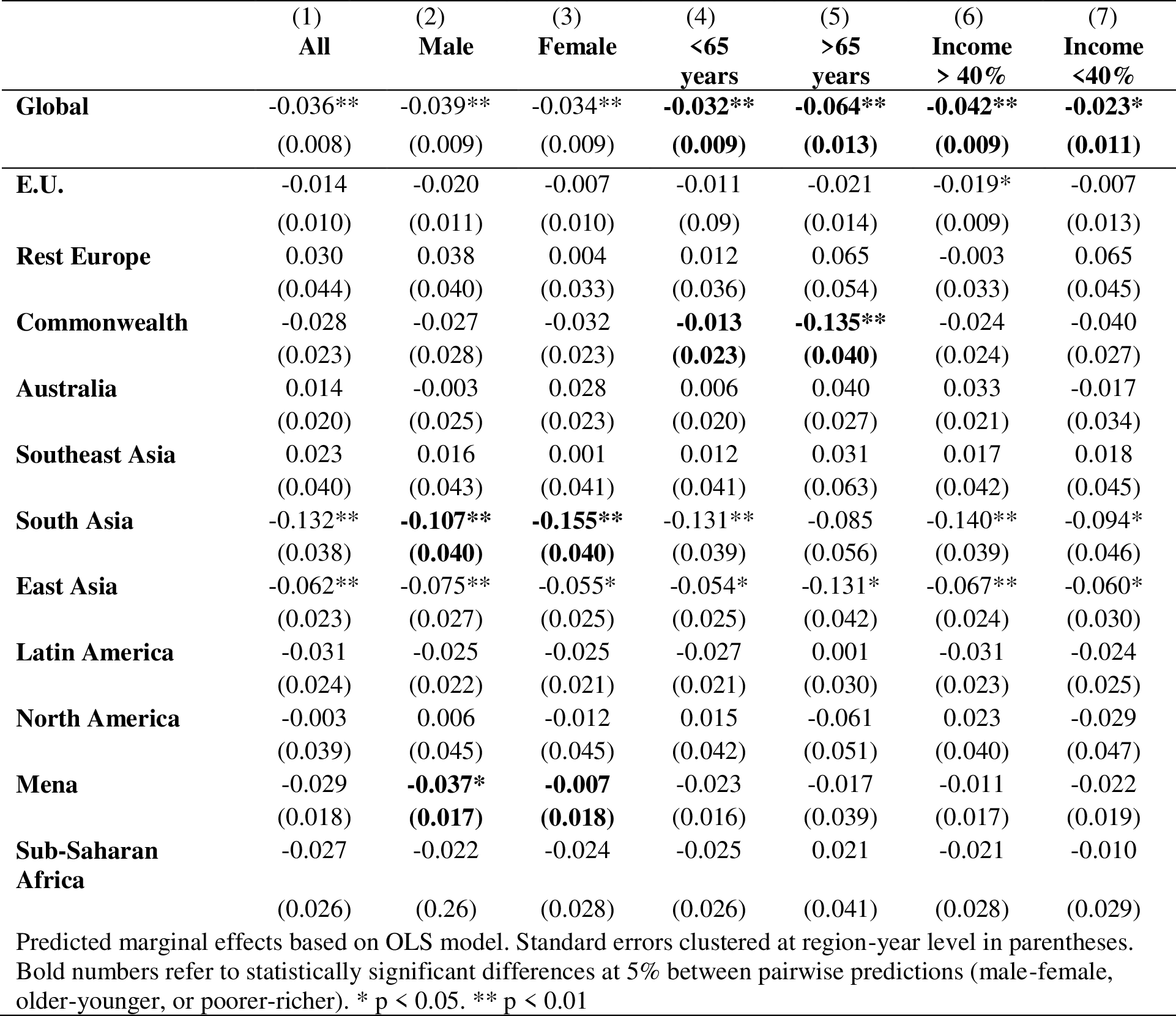

Supplement: S4 Table — Predicted marginal effects based on OLS model. Standard errors clustered at region-year level in parentheses. Bold numbers refer to statistically significant differences at 5% between pairwise predictions (male-female, older-younger, or poorer-richer). * p < 0.05. ** p < 0.01. (TIF) [file pone.0299983.s011.tif]

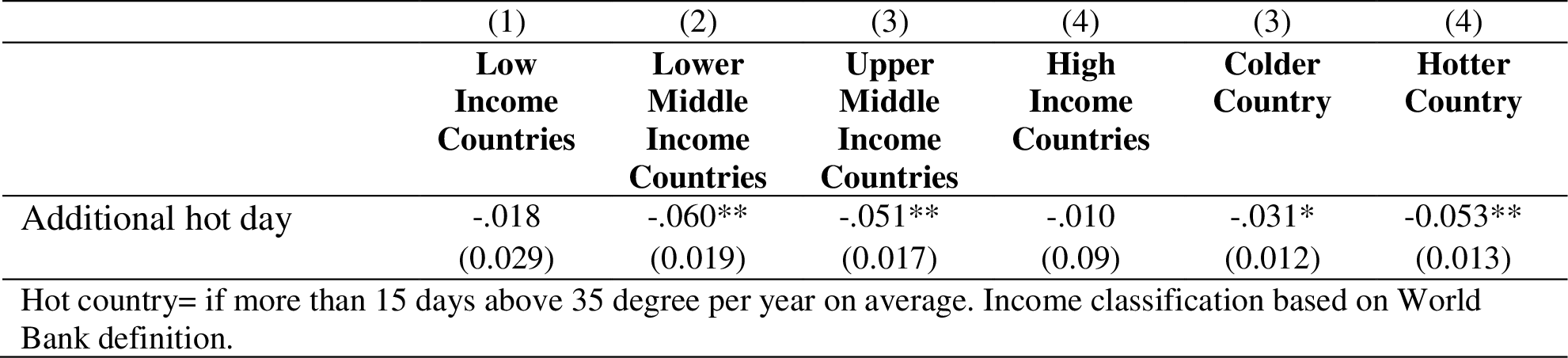

Supplement: S5 Table — (TIF) [file pone.0299983.s012.tif]

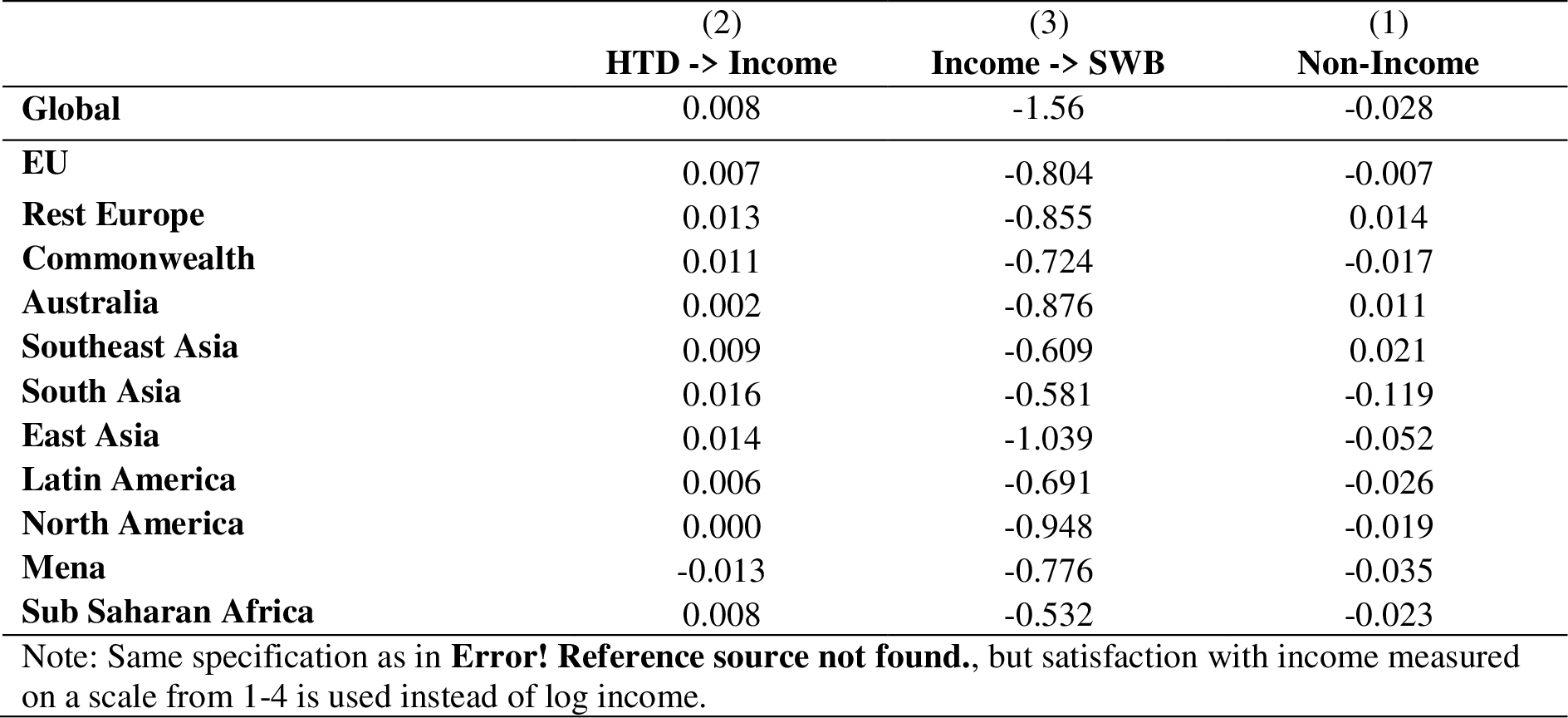

Supplement: S6 Table — (TIF) [file pone.0299983.s013.tif]
